# Assessing Clinicians’ Documentation of Vision in Older Adults Who Presented With a Fall at the Accident and Emergency Department of Northampton General Hospital

**DOI:** 10.7759/cureus.73183

**Published:** 2024-11-06

**Authors:** Evdokia Sourla, Michael Blumenthal Yohai, Khalid Ismail

**Affiliations:** 1 Ophthalmology, Birmingham and Midland Eye Centre, Birmingham, GBR; 2 Accident and Emergency, Northampton General Hospital, National Health Service Trust, Northampton, GBR

**Keywords:** clinical audit, elderly, head injury, vision examination, visual impairment

## Abstract

Background

Falls among elderly adults are one of the most common reasons that could lead to injury and modality, as vision is one of the modifiable risk factors for falls. By assessing it, we can detect those needing further follow-up with opticians or ophthalmologists, lowering the risk of falls secondary to poor vision.

Methods

Data were collected and reviewed retrospectively from a consecutive list of patients who presented with a fall or head injury to the Accident and Emergency Department at Northampton General Hospital. A total of 180 patients aged 75 years or older were randomly selected between November 2022 and January 2023. This audit measured the vision documentation in the vision assessment tool used in the Emergency Department at Northampton General Hospital and was based on the National Institute for Health and Clinical Excellence (NICE) and the Royal College of Physicians guidelines.

Results

Out of 180 patients in the sample, 34 (19%) had their visual assessments documented. Among them, around six (17.6%) out of 34 patients had a full vision assessment documenting all the sections in the vision assessment tool. Five (14.7%) out of 34 patients and 11 (32.3%) out of 34 patients did not have documentation about their distance and near vision, respectively. The visual fields were not documented in 22 (64.7%) out of 34 patients, and no assessment of the eye movements was reported in 16 (47%) out of 34 patients.

Discussion

Most of the patients in the Emergency Department lacked visual documentation, resulting in low compliance with the standards. One of the factors that contribute to elderly people's falling is low vision. Uncorrected refractive errors are one of the main causes of poor vision, but their correction is also associated with an increased risk of falls among elderly patients, as they require more time to adapt to changes in prescription (e.g., new glasses). In addition, patients who suffer from some eye conditions, such as glaucoma or macular degeneration, also have a high incidence of falls caused by an impairment of the visual fields.

Conclusions

All patients over 75 years old who presented with a fall to the Emergency Department should have a vision assessment. Vision documentation is essential to identify patients with vision impairment needing to receive an eye assessment after their discharge to reduce the risk of falls derived from poor vision. Strategies to improve this include training and the dissemination of information (for example, posters), which could help increase documentation rates.

## Introduction

Falls are one of the frequent causes of injury-related morbidity and mortality among older adults [1]. It has been reported that half of the adults over 80 years old will experience at least one fall annually, while one-third of adults who are 65 years old will have a fall each year [2]. Among the elderly people who experience a fall, around 20% of them will sustain severe injuries such as fractures or head injuries [3]. Falls could result in people limiting their activities and loss of their confidence and independence, which possibly results in reduced quality of life [4]. There are several scenarios that can cause a fall as a result of the interplay of various risk factors, such as muscular weakness, polypharmacy (defined as the use of four or more medications), poor balance, depression, orthostasis or dizziness, and low vision [5].

Vision impairment is one of the independent risk factors that can lead to people falling [6]. The reason for this situation is that people with poor vision may struggle to see objects and adapt their gait to avoid them or misjudge the position of step edges or lack of balance, which helps them to navigate the environment [6].

The main causes of poor vision in elderly people could be uncorrected refractive error, cataracts, glaucoma, and retinal degeneration [7]. These conditions could result in people losing their peripheral vision, depth perception, or contrast sensitivity [6]. Cataracts could affect contrast sensitivity and depth perception and restrict the visual field [8]. In addition, glaucoma could lead to reduced contrast sensitivity and limited visual fields [9]. Furthermore, wet age-related macular degeneration could lead not only to reduced vision but also to impaired contrast sensitivity [10].

Τhis article was previously presented as a poster at the 26^th^ Nottingham Eye Symposium & Research Meeting on January 19, 2024. 

## Materials and methods

Aim

The aim of this audit was to identify the assessment and documentation of vision in patients over 75 years old presenting with falls or head injuries at the Emergency Department of Northampton General Hospital, NHS Trust. The audit was conducted according to the National Institute of Clinical Excellence (NICE) guidelines, which mention that all patients who have a fall should undergo a multifactorial risk assessment, including a vision assessment, to reduce the incidence of falls [11]. Depending on the compliance of the clinicians with these standards, further actions would be applied to improve the holistic approach of patients over 75 years old who presented with a fall or head injury.

Objective

Patients detected with poor or impaired vision during assessment should be referred to optometrists or ophthalmologists for an eye review after their discharge from the hospital. Furthermore, patients who have not undergone eye examinations in the past year need a referral to the aforementioned specialists post-discharge. The primary objective was to identify patients who need further vision assessment aiming to prevent and reduce the incidence of falls caused by vision impairment.

Standards

Data were audited against the National Institute for Health and Clinical Excellence (NICE) guidelines and Clinical guideline (CG161) Falls in Older People: Assessing Risk and Prevention, which recommends a multifactorial assessment of patients who present with a fall [11].

The audit data were compared against the Royal College of Physicians’ tool of bedside vision assessment named ‘’Bedside Vision Check for fall prevention. Look out! ‘’, which was developed through a collaboration between the Royal College of Physicians, the British and Irish Orthoptic Society, the College of Optometrists, the Royal College of Ophthalmologists, the Royal College of Nursing, and NHS Improvement [12]. It mentions that a visual assessment needs to be conducted for all the patients presented with a fall.

Some of the parameters assessed by the tool include the ability to recognize objects from the end of the bed, which could be used as a screen for severe eyesight problems. Furthermore, the distance and near vision should be assessed for all patients, and, whenever possible, the visual field assessment and eye movements should be performed. If the eye examination must be stopped or is difficult to perform, possibly due to the patient’s condition, this should be recorded on the fall care plan as "vision not assessed," and the reason should be clearly stated [12].

The Emergency Department at Northampton General Hospital utilized the “Falls Care Bundle” for patients over 75 years old presenting with falls. The bundle includes a section on vision assessment, which is illustrated in Appendices (Figure 8) and is based on guidance from the Royal College of Physicians [12]. The vision assessment tool includes two images, refer to Appendices (Figures 9, 10), from the Royal College of Physicians ‘’the Bedside Vision Check for Fall Prevention Look out! ‘’, which are used to check patients’ distance and near vision, respectively [12].

Distant vision assessment could be achieved by placing the image, which is illustrated in Appendices (Figure 9), two meters away from the patient's bed and asking the patient to read the picture or sentences on it [12]. To assess near vision the patient is asked to put himself in a comfortable position for them (bent arm’s length) to read or recognize the image, which is presented in Appendices (Figure 10) [12]. A ''normal'' distance and near vision assessment is defined by the ability of the patient to fully and correctly read the images, which are illustrated in Appendices (Figures 9, 10). An ''abnormal'' assessment is characterized by the inability of the patient to read the images, which are demonstrated in Appendices (Figures 9, 10).

Data collection

The data represented the first-cycle audit using retrospective data collected by reviewing the clinical notes and the scanned vision assessment tool, which is shown in Appendices (Figure 8), of 180 patients over 75 years old presented to Northampton General Hospital Emergency Department with falls or head injuries between November 2022 and January 2023.

Data was collected randomly using a consecutive list, from which 180 patients were selected (approximately 60 patients from each month) on different days. This data was obtained from the Symphony system (EMIS Health, Leeds UK), which is a clinical system used for patient management, documentation, and tracking in the Emergency Department at Northampton General Hospital.

Audit criteria

Inclusion criteria required patients to be over 75 years old and attending due to falls or head injury in the Emergency Department at Northampton General Hospital. No exclusion criteria were reported.

Data analysis

Data was collected from the symphony system (EMIS Health, Leeds UK) and analyzed using a Microsoft Excel document (Microsoft Corporation, 2008) by Khalid Ismail.

## Results

Records of 180 patients were reviewed to establish whether a vision assessment had been conducted and documented either in the notes or the vision assessment tool, which is illustrated in Appendices (Figure 8). The primary characteristic of the sample included an average patient age of 83.88 years old (range of 75-95), while there were almost equally shared sex distributions with a female-to-male ratio of 3:2 (Table 1). Furthermore, around 106 (58.9%) out of 180 patients did not have documented any details regarding their history of visual impairment (Table 1). Of the 180 patients, 74 (41.1%) had their history of visual impairment documented. Among these, 39 (21.7%) did not have a history of visual impairment, while 35 (19.4%) did. Additionally, 13 (7.2%) of the 180 patients reported suffering from refractive errors, while the reason for poor vision was not reported for 16 (8.8%) of them (Table 1).

**Table 1:** Baseline characteristics of the studied sample n denotes the sample size. A total of 107 (59.4%) of the sample were female. Moreover, in 106 (58.9%) of the 180 patients, the history of vision impairment was not documented, whereas in 13 (7.2%) of the 180 patients, the reason for visual impairment was a refractive error. About two-thirds of the medical staff who assessed the patients were junior doctors. ACPs: advanced clinical practitioners

Gender (n=180)	
Male	73 (40.6%)
Female	107 (59.4%)
History of visual impairment (n=180)	
Not documented	106 (58.9%)
No history of visual impairment	39 (21.7%)
Blindness	2 (1.1%)
Error of refraction	13 (7.2%)
Cataract	2 (1.1%)
Macular degeneration	2 (1.1%)
Unknown/ not reported	16 (8.8%)
Grade of assessor (n=180)	
Junior doctors	119 (66.1%)
Middle-grade doctors	29 (16.1%)
ACPs	22 (12.2%)
Other	10 (5.6%)

Six (3.3%) of the 180 patients reported either blindness, cataracts, or macular degeneration, with approximately two (1.1%) reporting each condition (Table 1). Furthermore, junior doctors mostly contributed to the assessment and documentation of the patient’s vision, contributing to around 119 (66.1%) of the 180 documented cases (Table 1).

Visual assessment

**Figure 1:**
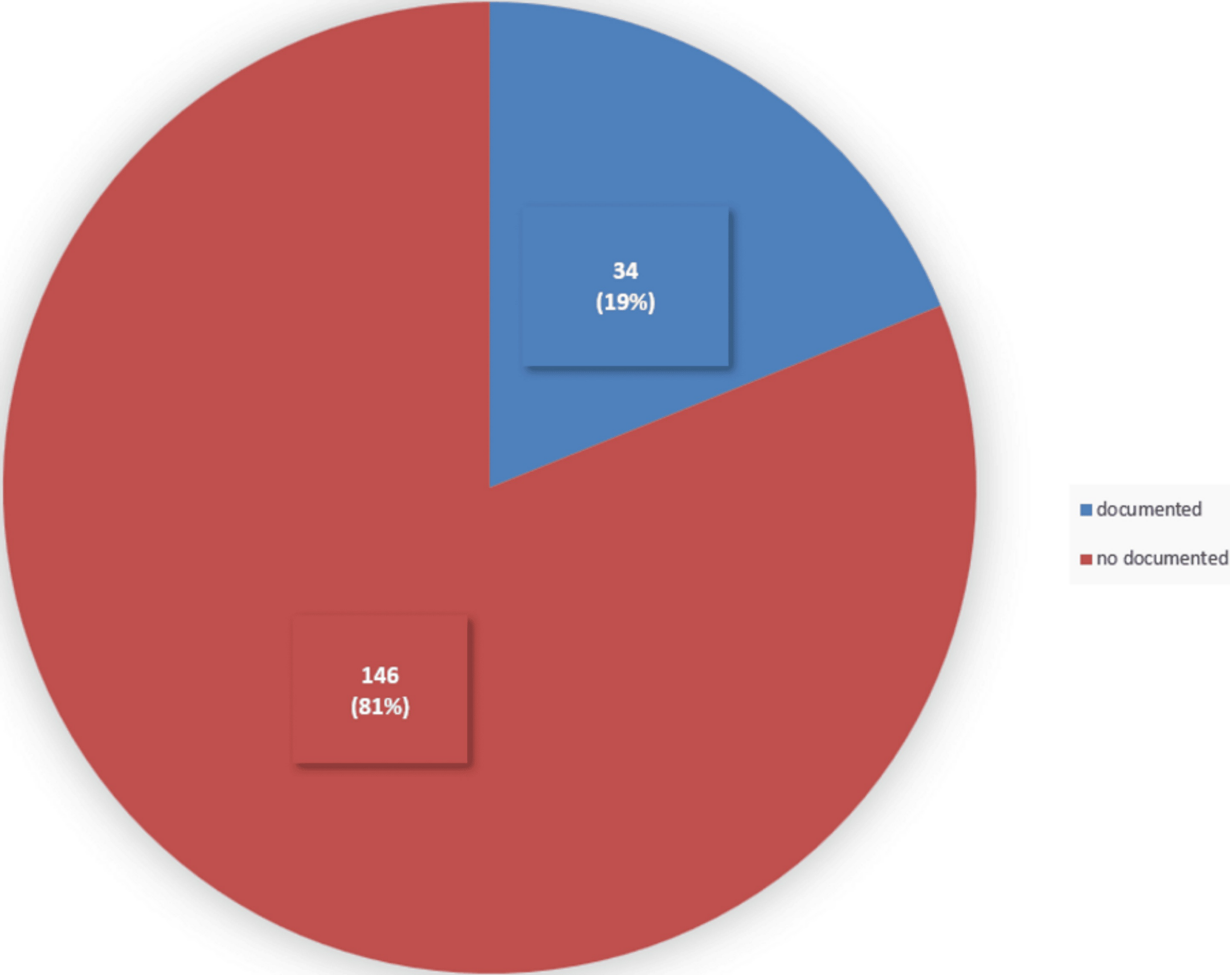
Frequency of visual assessment reporting among the studied sample A total of 34 (19%) of the 180 patients in the study sample had their visual assessment documented, while 146 (81%) did not.

Vision assessment documentation using the vision tool, which is demonstrated in the Appendices (Figure 8), showed that only 34 (19%) out of 180 patients in the sample had their visual assessment documented (Figure 1). These include the first three questions in the vision assessment tool, as shown in the Appendices (Figure 8), which ask whether the patient wears glasses, whether they have them with them, and when their last eye examination was.

**Figure 2:**
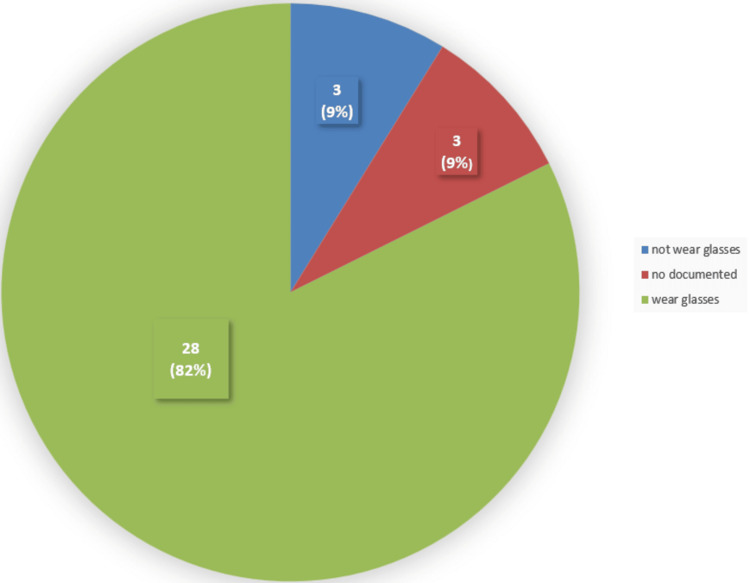
Number of patients who wore glasses among the studied sample A total of 28 (82%) of the 34 patients wore glasses, while three (9%) of the 34 patients did not wear glasses, and three (9%) of the 34 patients did not have this information documented.

Based on the results, it seems that most of the patients, around 28 (82%) out of 34 wore glasses, whereas three (9%) out of 34 patients did not wear glasses, and three (9%) of the 34 patients did not have documentation of it (Figure 2). Fifteen (53.6%) out of 28 patients carry them, while 13 (46.1%) of the 28 patients did not bring them. In addition, 28 (82%) of the 34 patients who wore glasses, 12 (42.8%) of 28 patients used them solely for reading, while 13 (46.4%) of 28 patients used them for everything. One patient reported using magnifying glasses and nothing was documented for another patient.

**Figure 3:**
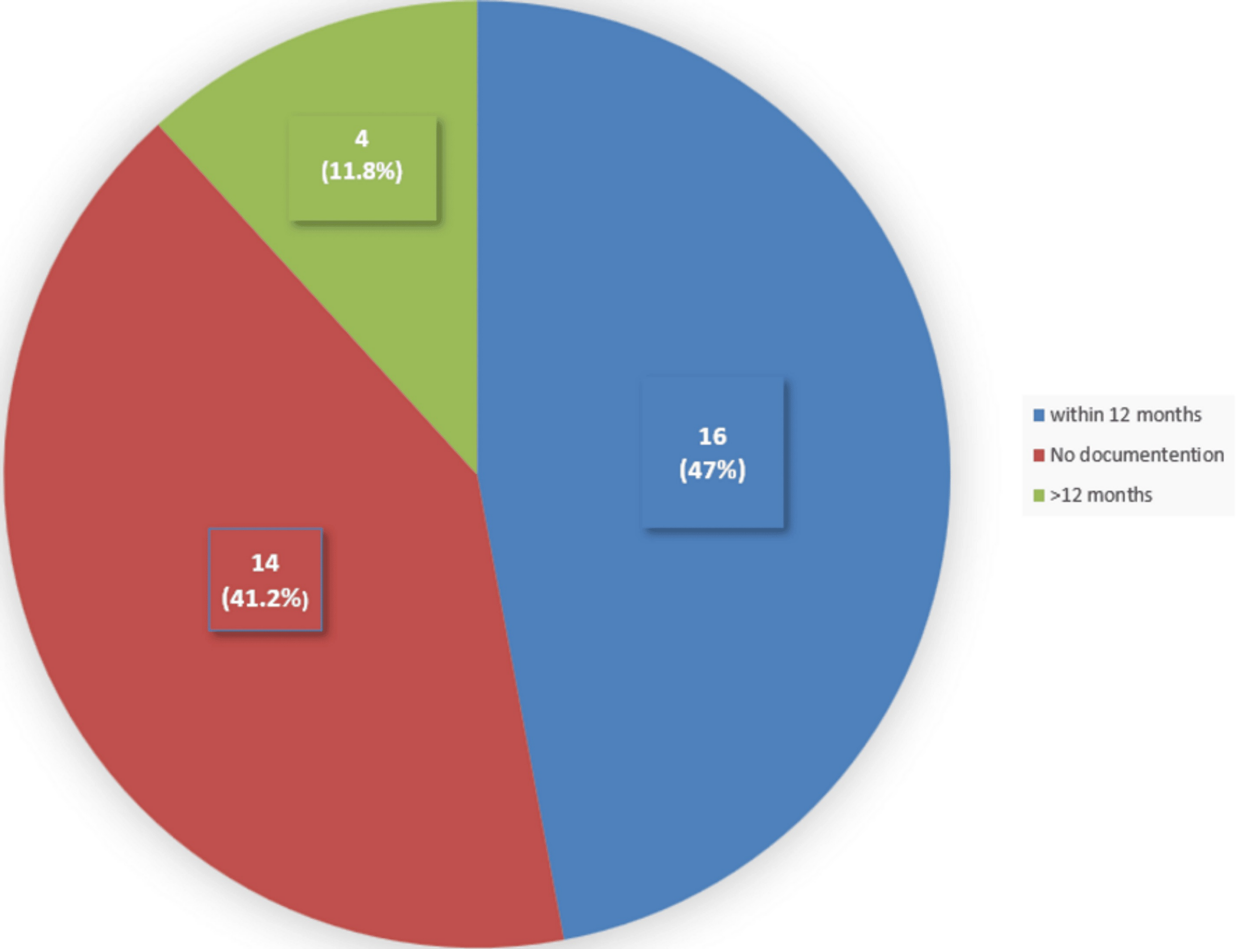
Frequency of last eye check among the studied sample Fourteen (41.2%) of the 34 patients did not have documentation of their last eye check. Sixteen (47%) of the 34 patients had their last eye check within 12 months of the onset of their fall. Four (11.8%) of the 34 patients had their last eye test more than one year ago.

A closer examination of the documentation revealed that 14 (41.2%) of 34 patients who underwent a visual assessment did not have information recorded about their most recent eye exam, 16 (47%) of 34 patients were found to have their last eye checked within 12 months from the date of their fall, and only four (11.8%) of 34 patients had their last eye test more than one year ago (Figure 3).

Details of visual assessment (distance and near vision)

**Figure 4:**
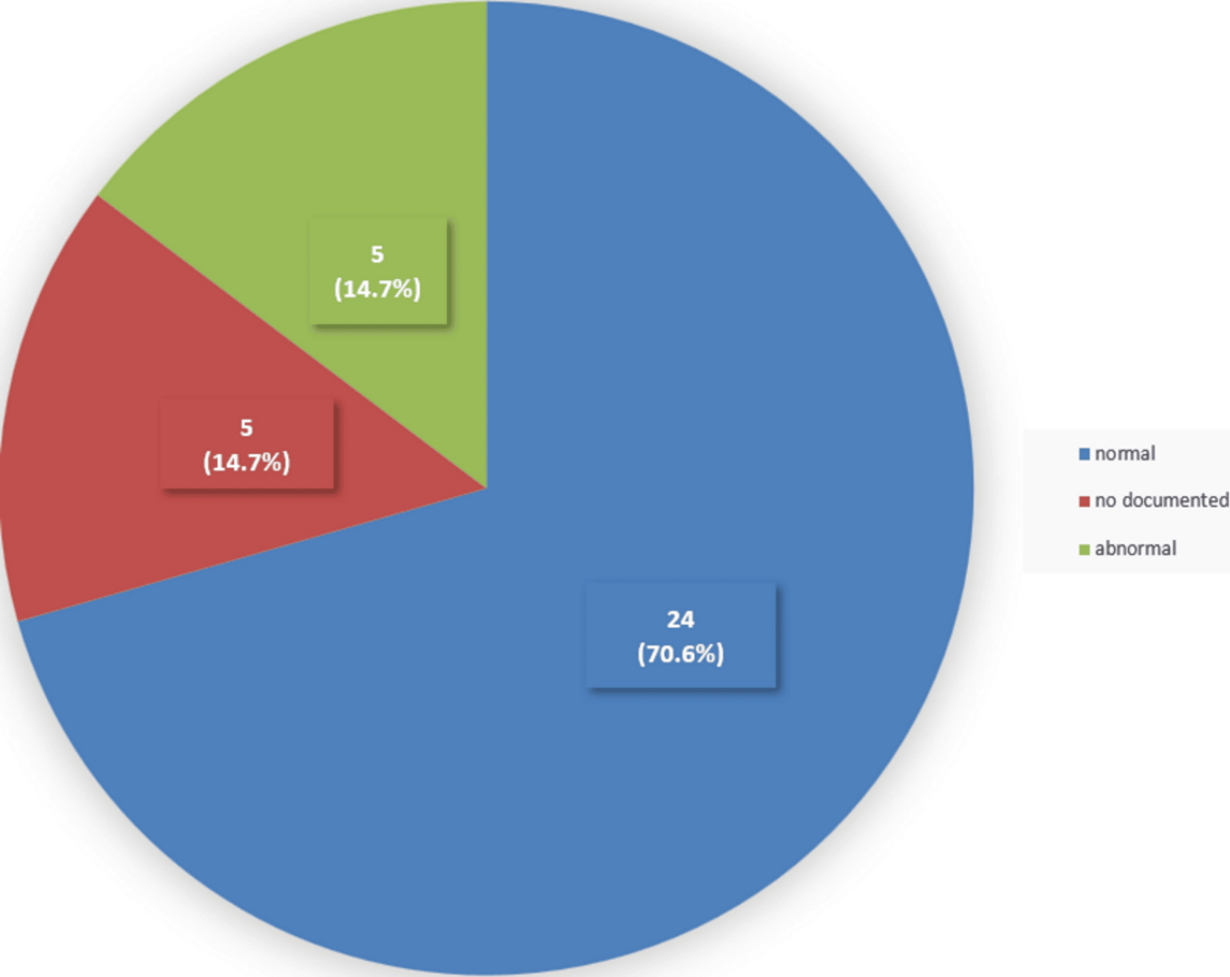
Distance vision assessment among the studied sample Five (14.7%) of the 34 patients did not have documentation of their distance vision. Twenty-four (70.6%) of the 34 patients were found to have normal distance vision, while five (14.7%) of the 34 patients had abnormal distance vision.

Looking at the data from the 34 patients who had the vision assessment documented, around five (14.7%) of them did not have any details about their distance vision (Figure 4). Twenty-nine (85.3%) of the 34 patients had their distance vision recorded. Of these, 24 (70.6%) of the 34 patients were found to have normal distance vision, while five (14.7%) of the 34 patients had abnormal distance vision (Figure 4).

**Figure 5:**
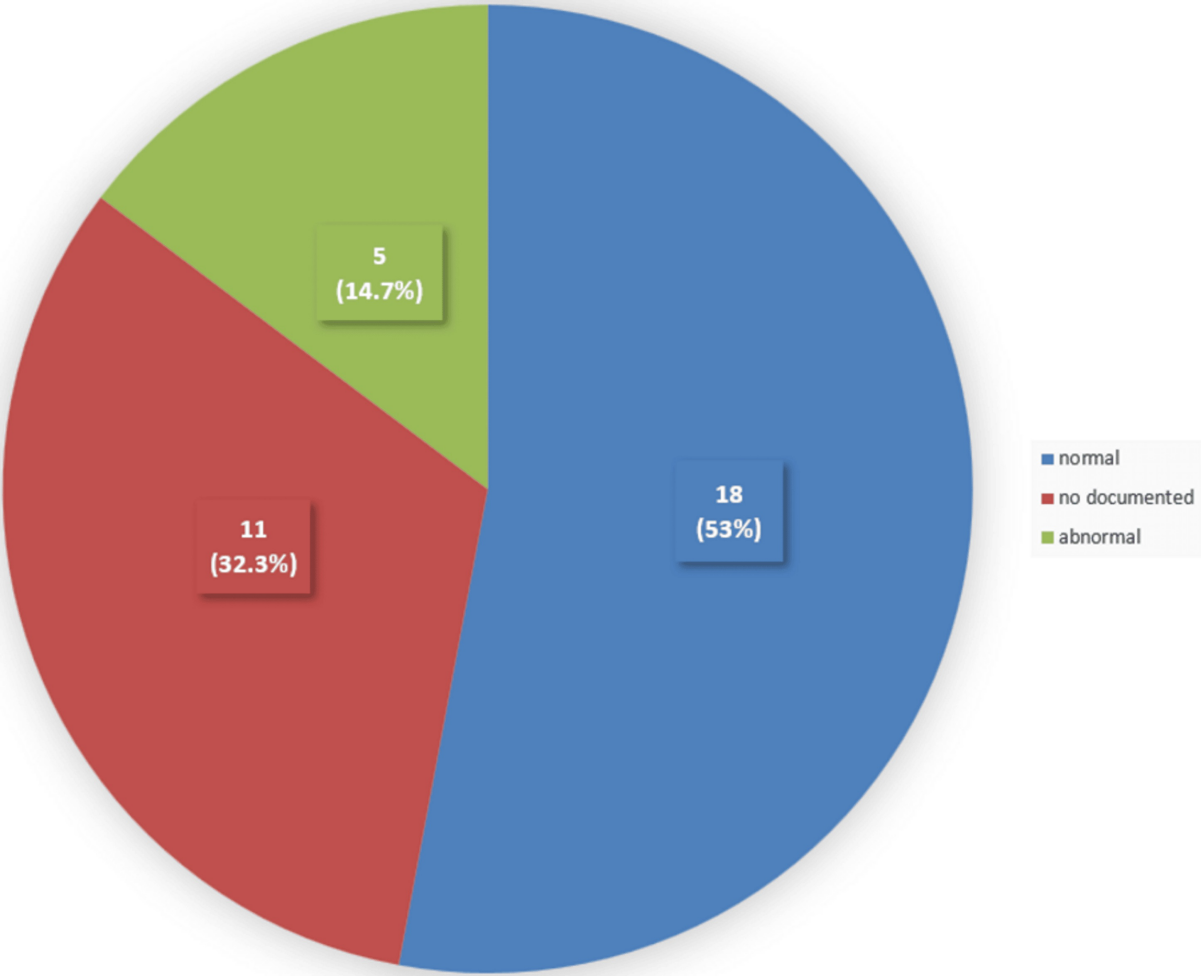
Near vision assessment among the studied sample Eleven (32.3%) of the 34 patients did not have documentation of their near vision. Eighteen (53%) of the 34 patients had normal near vision, while five (14.7%) of the 34 patients had abnormal near vision.

Regarding near vision, 11 (32.3%) of the 34 patients did not have a documented assessment (Figure 5). Eighteen (53%) of the 34 patients had their near vision recorded and were found to have normal vision, whereas five (14.7%) of the 34 patients were found to have abnormal near vision (Figure 5).

Details of visual assessment (visual field and eye movement)

**Figure 6:**
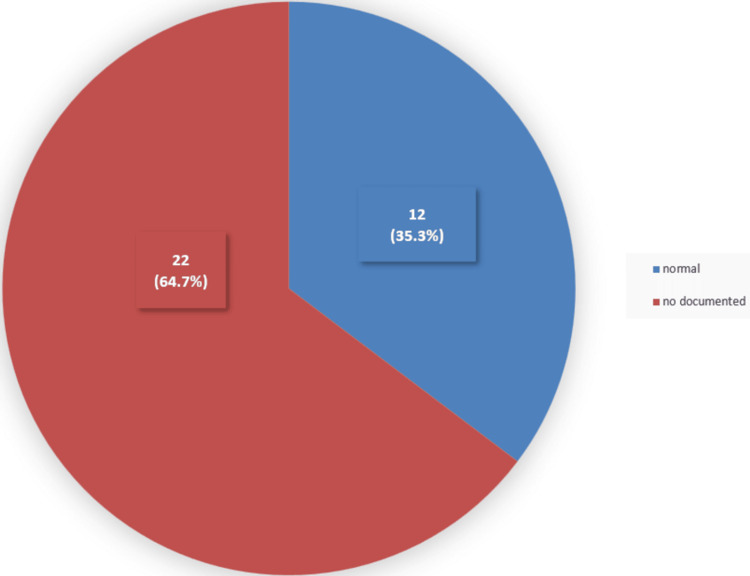
Visual field among the studied sample Twenty-two (64.7%) of the 34 patients did not have documentation of their visual fields. Twelve (35.3%) of the 34 patients had a normal visual field assessment.

Twenty-two (64.7%) of the 34 patients lacked a visual field assessment (Figure 6). All the patients who had their visual fields evaluated and documented had normal results (Figure 6).

**Figure 7:**
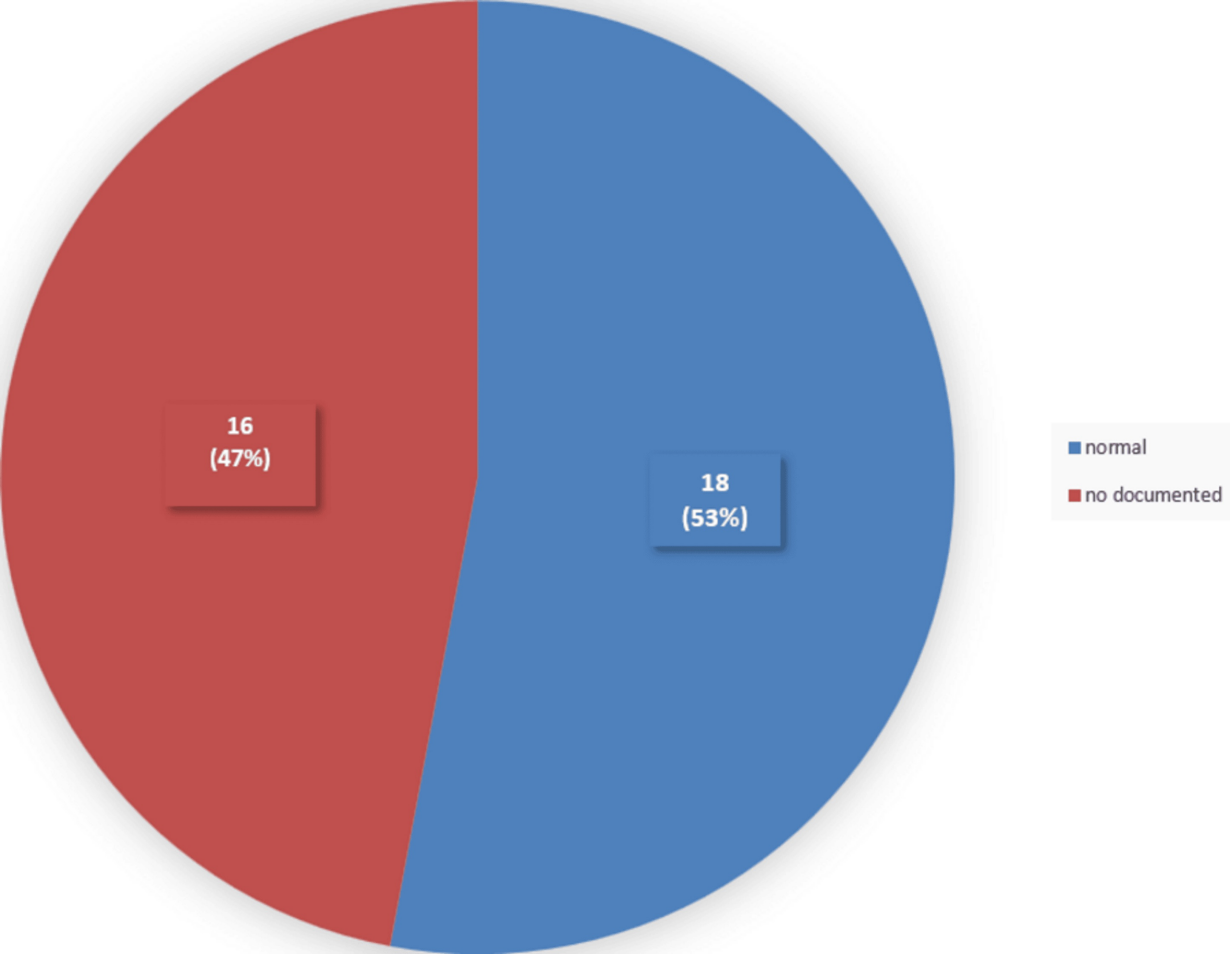
Eye movement among the studied sample Sixteen (47%) of the 34 patients did not have documentation of their eye movements. Eighteen (53%) of the 34 patients had normal eye movements.

Sixteen (47%) of 34 patients had not documented their eye movements, and the remaining 18 (53%) of 34 patients had unremarkable examination findings (Figure 7).

**Figure 8:**
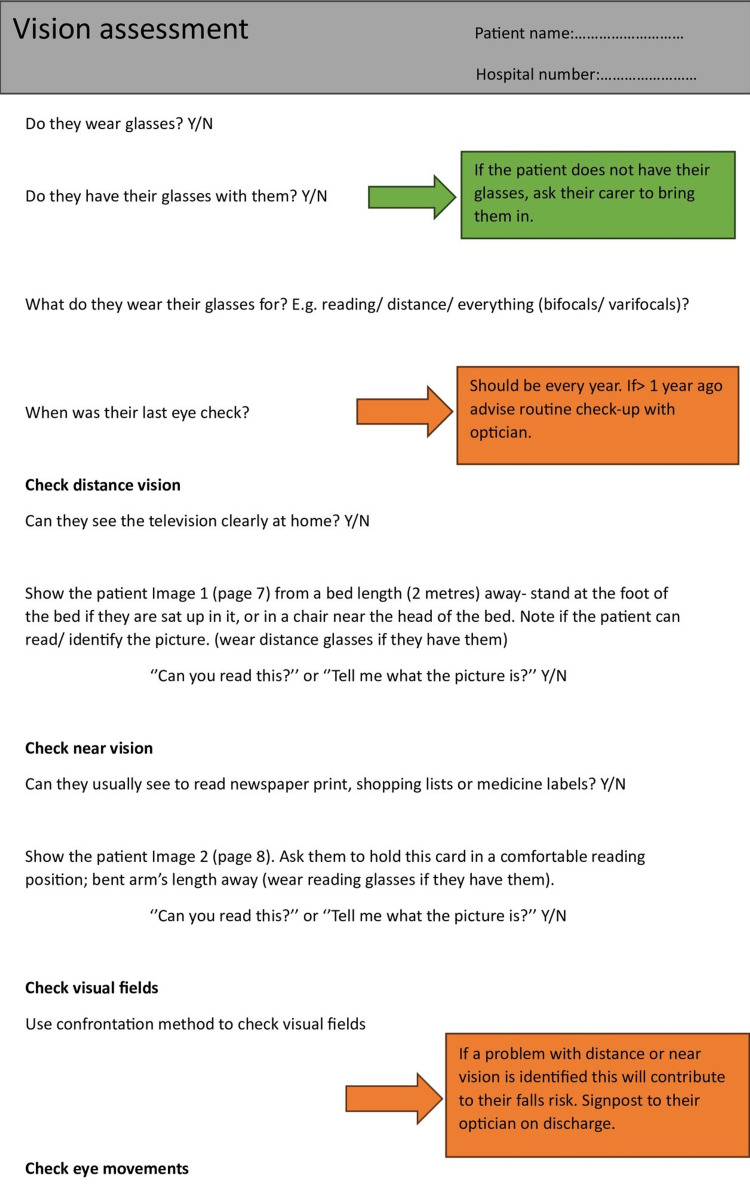
The bedside vision assessment tool in the Falls Care Bundle used in the Emergency Department, Northampton General Hospital

**Figure 9:**
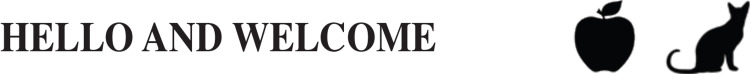
Image one of checking the distance vision of the patient by showing it from a beg length (2 m) away

**Figure 10:**
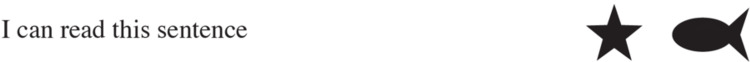
Image two of checking the near vision of the patient by asking them to hold it in their comfortable reading position (bent arm’s length)

Overall, only six (17.6%) of the 34 patients had a complete vision assessment, with all sections of the vision assessment tool (demonstrated in Appendices, Figure 8) filled in and documented by the clinicians. No documentation of the patients' vision assessment was found in their clinical notes. 

## Discussion

After completing the first audit cycle, we confirmed that clinicians including doctors or advanced clinical practitioners (ACPs) do not successfully document the patient’s vision as recommended by the NICE guidelines (CG161) Falls in Older People: Assessing Risk and Prevention and by the guide of the Royal College of Physicians [11,12]. Based on our hospital guidelines all patients over 75 years old should have a multifactorial assessment for the risk of falling.

Only 34 (19%) of 180 patients had their visual assessment documented, whereas only six (17.6%) of 34 patients had complete documentation using the vision tool illustrated in the Appendices (Figure 8). This is essential to identify patients with reduced vision acuity as it has been found to be closely associated with an increased rate of falls [13]. Moreover, none of the patients who did not have an eye examination documented the reason in the patient’s notes.

Additionally, out of the 180 patients, less than half of them 74 (41.1%) had documentation about whether they had a history of visual impairment. This information from the patient’s history could provide more details on whether they have any reflective errors, have undergone cataract surgery, or suffer from any eye conditions such as glaucoma or macular degeneration diseases. Based on the results, most of the patients, 28 (82%) of the 34, wore glasses, with 12 (42.8%) of the 28 patients using them for reading and 13 (46.4%) of the 28 patients using them for everything.

Refractive errors in elderly people, even with correction could increase the risk of falls due to the longer adaption of the change in the prescription that might occur among this population [14]. On the other hand, collecting data about whether the patient has undergone any cataract surgery is important, because it has been found that the first eye cataract surgery in an elderly woman can reduce the incidence of falls [15].

The results of the assessment of distance vision show that five (14.7%) out of 34 patients did not have documentation about distance vision, and five (14.7%) of 34 patients had abnormal findings. The records of near vision were less documented compared to distance vision, which was around 11 (32.3%) out of 34 patients. Among the patients who underwent near vision assessment, five (14.7%) of the 34 patients had abnormal findings. The abnormal distance or near vision findings at the examination are suggestive of being the cause of their fall. Further investigation of their eye disease or refractive errors is important to reduce the possibility of further falls due to poor vision.

There was no documentation about the visual fields of 22 (64.7%) of the 34 patients. This is essential to determine if a patient has a visual field deficit or is known to suffer from glaucoma or other macular degeneration diseases. According to Ouyang et al. (2022), these eye conditions increase the risk of falls. Patients who suffer from these eye conditions usually have slower walking speeds and loss of balance, which makes them prone to falling.

Around 14 (41.2%) of 34 patients did not have documentation about when was their last eye check, whereas four (11.8%) of 34 patients did not have their eyes checked for more than a year. This could be suggestive that a patient may have vision impairment, which has not been diagnosed. Clinicians should highlight the importance of annual checks [16].

Junior doctors mainly document the vision with the number of 119 (66.1%) vision assessments out of 180. One of the interventions that could help to improve the number of doctors who assess and document the patient’s vision is delivering teaching sessions to the clinicians. This teaching could be done at the regular teaching session for the junior doctors. These could include a video, which has been created by the Royal College of Physicians, showing how to assess a patient’s vision using the vision assessment tool [12]. Furthermore, teaching all clinicians including the Intermediate Care Team (ICT) may be useful as they assess patients with frequent falls to enhance their mobility and decrease chances of recurring falls. 

**Figure 11:**
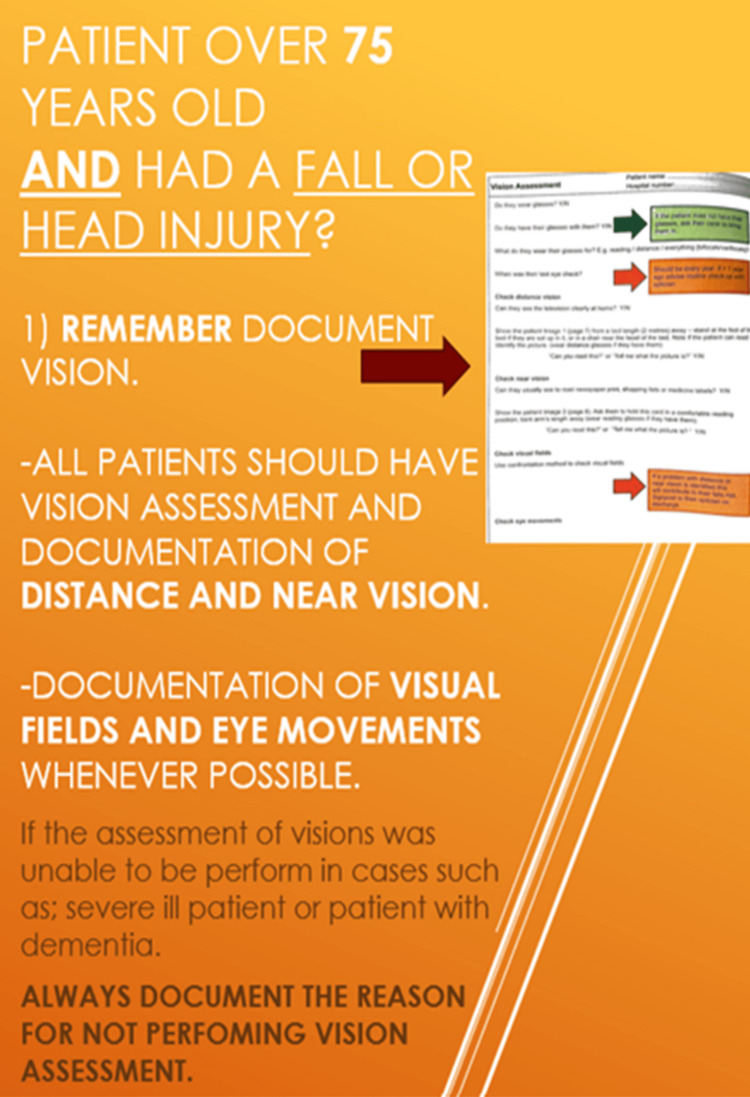
Proposed poster This could be placed in the doctor's office as a visible reminder for the assessment and documentation of patients' vision when they are over 75 years old and present with a fall or head injury.

Furthermore, one more way that could help the clinicians to be more compliant with the assessment and documentation of patients’ vision is by having posters in accessible locations and used by all clinicians who work in the Accident and Emergency department at Northampton General Hospital. This might act as a visible reminder to the doctors who are employed and work in the Accident and Emergency Department but also it would inform the locum doctors who are not fully aware of the hospital. An example of that is demonstrated in the Appendices (Figure 11).

Study limitations**

This study was limited by its retrospective nature, the absence of a control group, and a short period of study duration (November 2022 to January 2023). Unfortunately, at Northampton General Hospital the numerical scale of vision acuity is not used as it is challenging to be assessed for some patients, and the visual tool was implemented instead.

After analyzing the results of this audit, the intervention and the re-audit could not be performed as some people who were part of the team moved to another hospital. However, the results of this audit show that there is a significant lack of documentation for patients who are present with a fall or head injury in the Accident and Emergency department at Northampton General Hospital, NHS Trust.

Study strengths

The strengths of this study were the randomization of the sample, the large sample size, and alignment with established clinical standards, including the National Institute for Health and Care Excellence (NICE) guidelines (CG161) on Falls in Older People, and the Royal College of Physicians' vision assessment tool.

## Conclusions

The first audit cycle results showed that only a few patients have full documentation of their vision assessment, which shows that there is no good compliance with the standards recommendations from the NICE and the guidance by the Royal College of Physicians.

Teaching clinicians how to perform an eye examination and document a patient’s vision could improve documentation rates. Furthermore, teaching to all groups of practitioners is crucial to improve assessment and results. In addition, posters in the doctor’s office would be useful as a visible reminder. All these implementations could increase the assessment and documentation of patients’ vision, with the main aim not only to be compliant with the standards but also to identify patients who need a referral to a specialist to reduce their risk of falls due to poor vision.
